# New Sydenham Society’s Publications

**Published:** 1860-11

**Authors:** 


NEW SYDENHAM SOCIETY'S PUBLICATIONS.—In the July Number of the REVIEW, we mentioned that the works already issued by this society for the first year (1859) were five in number: 1. Diday, on Infantile Syphilis. 2. Gooch, on Diseases of Women. 3. Memoirs on Diphtheria, by Bretonneau and others. 4. Schrœder van der Kolk, on the Spinal Cord and Epilepsy. 5. Selected Monographs on Convulsions, Resections, and Iridectomy. We now add to this list the names of the books which members for 1860 will receive:—

Vol. VI.—Clinical Memoirs on Abdominal Tumors and Intumescence. By Dr. Bright. Collected and reprinted from the Guy's Hospital Reports. Edited by Dr. Barlow. With numerous wood-cuts.

Vol. VII.—A Year-Book for 1859 — The Year-Book is designed to form a register in condensed abstract of all important communications, whether British or Foreign, in Medicine, Surgery, and their allied Sciences during the year. It will consist of five sections:—1st. Anatomy and Physiology, edited by Dr. Harley. 2d. Medicine, edited by Dr. Handfield Jones. 3d. Surgery, edited by Mr. Hulke. 4th. Diseases of Women and Children, edited by Dr. Graily Hewitt. 5th. Forensic Medicine and Toxicology, edited by Dr. Odling.

Vol. VIII.—Frerichs's Clinical Account of Diseases of the Liver, vol. i. With wood-cuts. Translated by Dr. Murchison. (Already issued.)

Vol. IX.—The First Fasciculus of an Atlas of Illustrations of Diseases of the Skin, copied from those of Hebra.

Dr. Bright's Clinical Memoirs and the Year-Book will be ready for issue almost immediately. The Portraits of Skin Diseases will be three in number, and of life-size. They will, it is hoped, be ready in December.

The annual amount of subscription is *five dollars and a quarter*. The number of members has already reached nearly three thousand. The Honorary Local Secretaries for the United States and Canada, all of whom will be glad to receive the names and subscriptions of those wishing to become members, and to arrange for the regular distribution of their books, are as follows:—

*Philadelphia.*—Richard J. Dunglison, M.D., 121 South Tenth Street.

*Boston.*—R. H. Salter, M.D., 1 Stamford Street.

*New York.*—Charles F. Heywood, M.D., 66 West Twentieth Street.

*Montreal.*—Geo. E. Fenwick, M.D., 34 Little James Street.

